# Supplementary material for: Catastrophic costs for tuberculosis patients in India: Impact of methodological choices
Source: PLOS Glob Public Health. 2024 Apr 26;4(4):e0003078. doi: 10.1371/journal.pgph.0003078 (PMC11051603; doi:10.1371/journal.pgph.0003078)
Supplement: S1 Table — (DOCX) [file pgph.0003078.s001.docx]

**Table S1: Descriptive statistics of explanatory variables in regression analysis**

| Explanatory variables | General population (N = 528) | Urban slum dwellers (N = 526) | Tea garden families  (N = 403) | Combined (N = 1457) |
| --- | --- | --- | --- | --- |
| Percentage of TB affected households faced catastrophic cost using HCA1 at 20% threshold | 34 | 30 | 40 | 34 |
| Percentage of TB affected households faced catastrophic cost using HCA2 at 20% threshold | 32 | 30 | 35 | 32 |
| Percentage of TB affected households faced catastrophic cost using OA1 at 20% threshold | 59 | 56 | 62 | 59 |
| Percentage of pulmonary TB patients | 69 | 71 | 74 | 71 |
| Percentage of male patients | 66 | 59 | 59 | 61 |
| Average age of patients, years, Mean (SD) | 40 (15) | 38 (14) | 34 (13) | 38 (14) |
| Percentage of patients with up to primary education | 37 | 32 | 72 | 45 |
| Percentage of patients with secondary education | 32 | 46 | 20 | 34 |
| Percentage of patients with higher secondary education and above | 31 | 22 | 8 | 21 |
| Percentage of patients with income less than INR 100,000 | 25 | 24 | 59 | 34 |
| Percentage of patients with income from INR 100,000 to less than INR 200,000 | 36 | 42 | 41 | 40 |
| Percentage of patients with income INR 200,000 and above | 39 | 33 | --- | 26 |
| Average duration from symptom onset to treatment initiation, weeks, Mean (SD) | 9 (9) | 9 (9) | 7 (8) | 9 (9) |
| Percentage of patients with health insurance | 28 | 21 | 34 | 27 |
| Average direct cost of TB treatment, INR, Mean (SD) | 22740 (31905) | 18278 (29824) | 8312 (10073) | 17149 (27397) |
| Percentage of urban population | 52 | 100 | 0 | 55 |
