# Supplementary material for: Catastrophic costs for tuberculosis patients in India: Impact of methodological choices
Source: PLOS Glob Public Health. 2024 Apr 26;4(4):e0003078. doi: 10.1371/journal.pgph.0003078 (PMC11051603; doi:10.1371/journal.pgph.0003078)
Supplement: S2 Table — (DOCX) [file pgph.0003078.s002.docx]

**Table S2: Likelihood of incurring catastrophic cost using HCA1 method of indirect cost calculation**

|  | General population (N=528) | | Urban slum dwellers  (N=526) | | Tea garden families (N=403) | | All participants  (N=1457) | |
| --- | --- | --- | --- | --- | --- | --- | --- | --- |
| Explanatory variables | Unadjusted OR (95% CI) | p-value | Unadjusted OR (95% CI) | p-value | Unadjusted OR (95% CI) | p-value | Unadjusted OR (95% CI) | p-value |
| **Age (in years)** | 1.02  (1.00, 1.03) | 0.01 | 1.00  (0.99, 1.02) | 0.52 | 1.01  (0.99, 1.03) | 0.18 | 1.01  (1.00, 1.02) | 0.01 |
| **Sex** |  |  |  |  |  |  |  |  |
| Male (Reference) | 1.00 |  | 1.00 |  | 1.00 |  | 1.00 |  |
| Female | 1.24  (0.86, 1.80) | 0.25 | 2.26  (1.54, 3.30) | <0.001 | 1.47  (0.98, 2.21) | 0.06 | 1.57  (1.26, 1.95) | <0.001 |
| **Education** |  |  |  |  |  |  |  |  |
| Up to primary education (Reference) | 1.00 |  | 1.00 |  | 1.00 |  | 1.00 |  |
| Secondary education | 0.85  (0.56, 1.30) | 0.46 | 0.63  (0.41, 0.97) | 0.04 | 1.22  (0.74, 2.01) | 0.42 | 0.77  (0.60, 0.98) | 0.03 |
| Higher secondary education and above | 0.44  (0.28, 0.69) | <0.001 | 0.84  (0.51, 1.39) | 0.51 | 0.63  (0.28, 1.41) | 0.26 | 0.60  (0.45, 0.80) | <0.001 |
| **Pre-TB annual household income (Indian Rupee)** |  |  |  |  |  |  |  |  |
| Less than 100,000 (Reference) | 1.00 |  | 1.00 |  | 1.00 |  | 1.00 |  |
| 100,000 – less than 200,000 | 0.22  (0.14, 0.36) | <0.001 | 0.30  (0.19, 0.48) | <0.001 | 0.29  (0.19, 0.45) | <0.001 | 0.30  (0.23, 0.38) | <0.001 |
| 200,000 and above | 0.12  (0.07, 0.20) | <0.001 | 0.13  (0.08, 0.23) | <0.001 | --- |  | 0.17  (0.12, 0.23) | <0.001 |
| **Health insurance** |  |  |  |  |  |  |  |  |
| Having health insurance (Reference) | 1.00 |  | 1.00 |  | 1.00 |  | 1.00 |  |
| Not having health insurance | 1.13  (0.76, 1.70) | 0.55 | 1.03  (0.65, 1.64) | 0.90 | 0.95  (0.62, 1.44) | 0.79 | 0.99  (0.78, 1.25) | 0.91 |
| **Type of TB** |  |  |  |  |  |  |  |  |
| Pulmonary TB (Reference) | 1.00 |  | 1.00 |  | 1.00 |  | 1.00 |  |
| Extrapulmonary TB | 1.61  (1.10, 2.34) | 0.01 | 2.86  (1.92, 4.27) | <0.001 | 1.13  (0.72, 1.78) | 0.59 | 1.77  (1.40, 2.23) | <0.001 |
| **Delay from symptom initiation to treatment** | 1.01  (1.00, 1.03) | 0.13 | 1.04  (1.02, 1.06) | <0.001 | 1.02  (0.99, 1.04) | 0.15 | 1.02  (1.01, 1.04) | <0.001 |
| **Direct cost of TB treatment (Log cost)** | 2.10  (1.71, 2.58) | <0.001 | 4.41  (3.23, 6.01) | <0.001 | 1.42  (1.22, 1.66) | <0.001 | 1.79  (1.61, 1.99) | <0.001 |
| **Residential status** |  |  |  |  |  |  |  |  |
| Urban (Reference) | 1.00 |  | --- |  | --- |  | 1.00 |  |
| Rural | 1.14  (0.80, 1.61) | 0.48 |  |  |  |  | 1.39  (1.12, 1.72) | 0.00 |
| **Wealth quintile** |  |  |  |  |  |  |  |  |
| Poorest (Reference) | --- |  | --- |  | --- |  | --- |  |
| Poorer |  |  |  |  |  |  | 0.84  (0.61, 1.17) | 0.31 |
| Middle |  |  |  |  |  |  | 0.72  (0.51, 1.01) | 0.05 |
| Richer |  |  |  |  |  |  | 0.72  (0.52, 1.01) | 0.06 |
| Richest |  |  |  |  |  |  | 0.45  (0.32, 0.64) | <0.001 |

Notes: HCA1: Human capital approach where hours spent was calculated using minimum wage rate for all and household income as denominator; OR: Odds Ratio; CI: Confidence Interval; Blanks indicate Not Applicable.
