# Supplementary material for: Catastrophic costs for tuberculosis patients in India: Impact of methodological choices
Source: PLOS Glob Public Health. 2024 Apr 26;4(4):e0003078. doi: 10.1371/journal.pgph.0003078 (PMC11051603; doi:10.1371/journal.pgph.0003078)
Supplement: S3 Table — (DOCX) [file pgph.0003078.s003.docx]

**Table S3: Likelihood of incurring catastrophic cost using HCA2 method of indirect cost calculation**

|  | General population (N=528) | | Urban slum dwellers  (N=526) | | Tea garden families (N=403) | | All participants  (N=1457) | |
| --- | --- | --- | --- | --- | --- | --- | --- | --- |
| Explanatory variables | Unadjusted OR (95% CI) | p-value | Unadjusted OR (95% CI) | p-value | Unadjusted OR (95% CI) | p-value | Unadjusted OR (95% CI) | p-value |
| **Age (in years)** | 1.02  (1.01, 1.03) | 0.00 | 1.00  (0.99, 1.02) | 0.61 | 1.01  (0.99, 1.03) | 0.11 | 1.01  (1.00, 1.02) | 0.01 |
| **Sex** |  |  |  |  |  |  |  |  |
| Male (Reference) | 1.00 |  | 1.00 |  | 1.00 |  | 1.00 |  |
| Female | 1.26  (0.86, 1.84) | 0.23 | 2.30  (1.57, 3.36) | <0.001 | 1.65  (1.09, 2.51) | 0.02 | 1.68  (1.34, 2.10) | <0.001 |
| **Education** |  |  |  |  |  |  |  |  |
| Up to primary education (Reference) | 1.00 |  | 1.00 |  | 1.00 |  | 1.00 |  |
| Secondary education | 0.84  (0.55, 1.30) | 0.44 | 0.65  (0.42, 0.99) | 0.05 | 1.47  (0.89, 2.43) | 0.13 | 0.85  (0.66, 1.09) | 0.19 |
| Higher secondary education and above | 0.49  (0.31, 0.78) | 0.00 | 0.84  (0.51, 1.39) | 0.51 | 0.71  (0.31, 1.64) | 0.42 | 0.67  (0.50, 0.90) | 0.01 |
| **Pre-TB annual household income (Indian Rupee)** |  |  |  |  |  |  |  |  |
| Less than 100,000 (Reference) | 1.00 |  | 1.00 |  | 1.00 |  | 1.00 |  |
| 100,000 – less than 200,000 | 0.26  (0.16, 0.42) | <0.001 | 0.31  (0.20, 0.49) | <0.001 | 0.36  (0.23, 0.57) | <0.001 | 0.35  (0.27, 0.45) | <0.001 |
| 200,000 and above | 0.08  (0.05, 0.14) | <0.001 | 0.13  (0.08, 0.23) | <0.001 | --- |  | 0.14  (0.10, 0.20) | <0.001 |
| **Health insurance** |  |  |  |  |  |  |  |  |
| Having health insurance (Reference) | 1.00 |  | 1.00 |  | 1.00 |  | 1.00 |  |
| Not having health insurance | 0.92  (0.61, 1.37) | 0.67 | 1.04  (0.66, 1.65) | 0.86 | 0.96  (0.62, 1.49) | 0.87 | 0.95  (0.74, 1.21) | 0.67 |
| **Type of TB** |  |  |  |  |  |  |  |  |
| Pulmonary TB (Reference) | 1.00 |  | 1.00 |  | 1.00 |  | 1.00 |  |
| Extrapulmonary TB | 1.66  (1.13, 2.44) | 0.01 | 2.82  (1.89, 4.20) | <0.001 | 1.26  (0.79, 1.99) | 0.33 | 1.85  (1.46, 2.34) | <0.001 |
| **Delay from symptom initiation to treatment** | 1.02  (1.00, 1.04) | 0.04 | 1.04  (1.02, 1.06) | <0.001 | 1.02  (0.99, 1.04) | 0.15 | 1.03  (1.01, 1.04) | <0.001 |
| **Direct cost of TB treatment (Log cost)** | 3.84  (2.90, 5.09) | <0.001 | 4.46  (3.26, 6.09) | <0.001 | 1.66  (1.39, 1.98) | <0.001 | 2.28  (2.01, 2.59) | <0.001 |
| **Residential status** |  |  |  |  |  |  |  |  |
| Urban (Reference) | 1.00 |  | --- |  | --- |  | 1.00 |  |
| Rural | 1.01  (0.70, 1.45) | 0.97 |  |  |  |  | 1.13  (0.91, 1.41) | 0.27 |
| **Wealth quintile** |  |  |  |  |  |  |  |  |
| Poorest (Reference) | --- |  | --- |  | --- |  | 1.00 |  |
| Poorer |  |  |  |  |  |  | 0.94  (0.67, 1.32) | 0.73 |
| Middle |  |  |  |  |  |  | 0.78  (0.55, 1.09) | 0.15 |
| Richer |  |  |  |  |  |  | 0.81  (0.57, 1.13) | 0.21 |
| Richest |  |  |  |  |  |  | 0.42  (0.29, 0.61) | <0.001 |

Notes: HCA2: Human capital approach where hours spent was calculated using combination of patient wage and minimum wage and household income as denominator; OR: Odds Ratio; CI: Confidence Interval; Blanks indicate Not Applicable.
