# Supplementary material for: Catastrophic costs for tuberculosis patients in India: Impact of methodological choices
Source: PLOS Glob Public Health. 2024 Apr 26;4(4):e0003078. doi: 10.1371/journal.pgph.0003078 (PMC11051603; doi:10.1371/journal.pgph.0003078)
Supplement: S4 Table — (DOCX) [file pgph.0003078.s004.docx]

**Table S4: Likelihood of incurring catastrophic cost using OA1 method of indirect cost calculation**

|  | General population (N=528) | | Urban slum dwellers  (N=526) | | Tea garden families (N=403) | | All participants  (N=1457) | |
| --- | --- | --- | --- | --- | --- | --- | --- | --- |
| Explanatory variables | Unadjusted OR (95% CI) | p-value | Unadjusted OR (95% CI) | p-value | Unadjusted OR (95% CI) | p-value | Unadjusted OR (95% CI) | p-value |
| **Age (in years)** | 1.01  (1.00, 1.02) | 0.14 | 1.01  (1.00, 1.02) | 0.12 | 1.01  (1.00, 1.03) | 0.09 | 1.01  (1.00, 1.02) | 0.01 |
| **Sex** |  |  |  |  |  |  |  |  |
| Male (Reference) | 1.00 |  | 1.00 |  | 1.00 |  | 1.00 |  |
| Female | 0.49  (0.34, 0.71) | <0.001 | 0.93  (0.66, 1.32) | 0.69 | 0.69  (0.46, 1.04) | 0.07 | 0.69  (0.56, 0.85) | <0.001 |
| **Education** |  |  |  |  |  |  |  |  |
| Up to primary education (Reference) | 1.00 |  | 1.00 |  | 1.00 |  | 1.00 |  |
| Secondary education | 0.70  (0.46, 1.07) | 0.10 | 0.75  (0.50, 1.13) | 0.17 | 1.51  (0.89, 2.55) | 0.13 | 0.84  (0.66, 1.07) | 0.16 |
| Higher secondary education and above | 0.49  (0.32, 0.75) | 0.00 | 0.70  (0.43, 1.13) | 0.15 | 1.06  (0.50, 2.28) | 0.87 | 0.66  (5.00, 0.86) | 0.00 |
| **Pre-TB annual household income (Indian Rupee)** |  |  |  |  |  |  |  |  |
| Less than 100,000 (Reference) | 1.00 |  | 1.00 |  | 1.00 |  | 1.00 |  |
| 100,000 – less than 200,000 | 0.55  (0.34, 0.88) | 0.01 | 0.56  (0.35, 0.90) | 0.01 | 1.29  (0.85, 1.94) | 0.28 | 0.79  (0.61, 1.01) | 0.06 |
| 200,000 and above | 0.40  (0.25, 0.64) | <0.001 | 0.34  (0.21, 0.54) | <0.001 | --- |  | 0.48  (0.37, 0.63) | <0.001 |
| **Health insurance** |  |  |  |  |  |  |  |  |
| Having health insurance (Reference) | 1.00 |  | 1.00 |  | 1.00 |  | 1.00 |  |
| Not having health insurance | 0.73  (0.49, 1.08) | 0.12 | 0.92  (0.60, 1.40) | 0.68 | 0.76  (0.50, 1.17) | 0.22 | 0.78  (0.61, 0.99) | 0.04 |
| **Type of TB** |  |  |  |  |  |  |  |  |
| Pulmonary TB (Reference) | 1.00 |  | 1.00 |  | 1.00 |  | 1.00 |  |
| Extrapulmonary TB | 0.82  (0.56, 1.19) | 0.29 | 1.09  (0.74, 1.60) | 0.65 | 1.58  (0.98, 2.54) | 0.06 | 1.07  (0.85, 1.34) | <0.001 |
| **Delay from symptom initiation to treatment** | 1.00  (0.99, 1.02) | 0.65 | 1.03  (1.01, 1.05) | 0.01 | 1.02  (0.99, 1.05) | 0.19 | 1.02  (1.00, 1.03) | 0.01 |
| **Direct cost of TB treatment (Log cost)** | 1.69  (1.41, 2.02) | <0.001 | 1.64  (1.38, 1.96) | <0.001 | 1.30  (1.13, 1.48) | <0.001 | 1.37  (1.26, 1.49) | <0.001 |
| **Residential status** |  |  |  |  |  |  |  |  |
| Urban (Reference) | 1.00 |  | --- |  | --- |  | 1.00 |  |
| Rural | 1.23  (0.87, 1.75) | 0.24 |  |  |  |  | 1.26  (1.02, 1.55) | 0.03 |
| **Wealth quintile** |  |  |  |  |  |  |  |  |
| Poorest (Reference) | --- |  | --- |  | --- |  | 1.000 |  |
| Poorer |  |  |  |  |  |  | 1.11  (0.79, 1.55) | 0.55 |
| Middle |  |  |  |  |  |  | 1.26  (0.90, 1.77) | 0.18 |
| Richer |  |  |  |  |  |  | 0.92  (0.66, 1.29) | 0.64 |
| Richest |  |  |  |  |  |  | 0.62  (0.45, 0.86) | 0.00 |

Notes: OA1: Output approach with household income as denominator; OR: Odds Ratio; CI: Confidence Interval; Blanks indicate Not Applicable.
