# Supplementary material for: Catastrophic costs for tuberculosis patients in India: Impact of methodological choices
Source: PLOS Glob Public Health. 2024 Apr 26;4(4):e0003078. doi: 10.1371/journal.pgph.0003078 (PMC11051603; doi:10.1371/journal.pgph.0003078)
Supplement: S1 Data — (ZIP) [file pgph.0003078.s005.zip › 1st Round-Code Book.pdf]

Sheet name	Variable name	Code
Basic info	Sex	Male = 1; Female = 2
	Type of TB	Pulmonary, bacteriologically confirmed = 1<br>Pulmonary, bacteriologically unconfirmed = 2<br>Extra pulmonary = 3
	Patient's education	Not attended school = 1<br>Primary school = 2<br>Secondary school = 3<br>Higher secondary school = 4<br>Graduate = 5<br>Post-graduate and above = 6<br>Others = 7
	Residential status	Urban = 1<br>Rural = 2
	Outcome	Cure / treatment completion = 1<br>Relapse = 2<br>Death = 3<br>Defaulter = 4<br>Defaulter and relapse = 5<br>Treatment ongoing = 6
Visit	Type of provider	Pharmacy / Drugstore = 1<br>Dispensary = 2<br>Private clinic / private practitioner = 3<br>Quack = 4<br>Government health facility (health centres) = 5<br>Government hospital = 6<br>Traditional healers / herbalists = 7<br>AYUSH (Ayurveda, Yoga, Unani, Siddha, Homeopath) = 8<br>Private hospital = 9<br>Community health worker = 10<br>Diagnostic centre = 11<br>Others including TG hospital = 12
Hospitalization	Type of hospital	Government hospital / health centre = 1<br>NGO/charitable health centre or hospital = 2<br>Private hospital = 3<br>Others including TG hospital = 4
DOT	Medicine intake under supervision or not	Self-administered = 1<br>Directly observed treatment (DOT) = 2
	Who is the DOT provider?	Health facility = 1<br>Community health worker/volunteer = 2<br>Workplace = 3<br>Family member = 4<br>Others = 5
Picking up drug	Who goes to pick-up drugs?	Patient alone = 1<br>Patient with accompanying Person = 2<br>Household member = 3<br>Friend /neighbour = 4<br>Other = 5
	How often do they go for drug pick-up?	Every day = 1<br>Every week = 2

		Every 2 weeks = 3<br>Every month = 4<br>Other = 5
	Where do they go for drug pick-up?	Pharmacy/ Drugstore = 1<br>Dispensary = 2<br>Private clinic/ Private practitioner = 3<br>Quack = 4<br>Government health facility (CHC, PHC, SC) = 5<br>Government hospital = 6<br>Traditional healers/ Herbalists = 7<br>AYUSH (Ayurveda, Yoga, Unani, Siddha, Homeopath) = 8<br>Private hospital = 9<br>Community health worker = 10<br>Diagnostic centre = 11<br>Other including TG hospital = 12
Social position	Do you have health insurance?	Yes = 1; No = 2; Don't know = 3
	Type of health insurance	a. Employees state insurance scheme<br>b. Central government health scheme<br>c. State health insurance scheme<br>d. Rashtriya swasthya Bima Yojana<br>e. Community health insurance programme<br>f. Other health insurance through employer<br>g. Medical reimbursement from employer<br>h. Other privately purchased commercial health insurance<br>i. Other (specify)
	Main occupation	Unemployed = 1<br>Daily wage earner = 2<br>Service (private) = 3<br>Factory worker = 4<br>Business = 5<br>Farmer = 6<br>Government employee = 7<br>Teacher = 8<br>Retiree = 9<br>Homemaker = 10<br>Tea Garden worker = 11<br>Student = 12<br>Other = 13
	Primary employment	Unemployed = 1<br>Formal paid worker = 2<br>Informal paid worker = 3<br>Retired = 4<br>Student = 5<br>Housework = 6<br>Self-occupied = 7<br>Other = 8

	Source of drinking water	Pipe water = 1<br>Well = 2<br>Tubewell = 3<br>Bottled / river / spring water = 4<br>Others (specify) = 5
	Type of house	Pucca = 1<br>Semi pucca = 2<br>Kachha = 3
	Where the cooking is done	Outside the house (open area / semi open area) = 1<br>Separate kitchen = 2<br>Living / sleeping area = 3
	Cooking fuel	Electricity = 1<br>LPG cylinder = 2<br>Natural gas (gas pipeline) = 3<br>Biogas = 4<br>Kerosene = 5<br>Coal / Lignite = 6<br>Wood / Charcoal / agricultural crop waste = 7<br>Straw / grass / shrubs / scavenged twinges = 8<br>Dung cake = 9<br>No food cooked in household = 9<br>Other (specify) = 10
	Source of lighting	Electricity = 1<br>Biogas = 2<br>Solar = 3<br>Kerosene = 4<br>Other oil = 5<br>No lighting = 6<br>Other (specify) = 7
Coping	Did you borrow or receive any money to cover your TB expenses?	Yes = 1; No = 2; Don’t know = 3
	From whom did you borrow / receive money?	Family members / relatives = 1<br>Neighbours / friends / colleagues = 2<br>Commercial bank loan (public / private) = 3<br>Cooperative = 4<br>Employer = 5<br>“Unofficial lender” (Black market) = 6<br>Other (specify) = 7
	Are you expected to pay back the amount?	Yes = 1; No = 2; Don’t know = 3
	Have you sold / mortgaged any personal belongings for TB treatment?	Yes = 1; No = 2; Don’t know = 3
	What did you sell / mortgage?	Land = 1<br>Livestock = 2<br>Transport / vehicle = 3<br>Household items = 4<br>Farm product = 5

		Gold / jewellery = 6<br>Other (specify) = 7
