# Supplementary material for: Catastrophic costs for tuberculosis patients in India: Impact of methodological choices
Source: PLOS Glob Public Health. 2024 Apr 26;4(4):e0003078. doi: 10.1371/journal.pgph.0003078 (PMC11051603; doi:10.1371/journal.pgph.0003078)
Supplement: S1 Data — (ZIP) [file pgph.0003078.s005.zip › 2nd Round-Code Book.pdf]

Sheet name	Variable name	Code
Basic info	Sex	Male = 1; Female = 2
Visit	Type of provider	Pharmacy / Drugstore = 1<br>Dispensary = 2<br>Private clinic / private practitioner = 3<br>Quack = 4<br>Government health facility (health centres) = 5<br>Government hospital = 6<br>Traditional healers / herbalists = 7<br>AYUSH (Ayurveda, Yoga, Unani, Siddha, Homeopath) = 8<br>Private hospital = 9<br>Community health worker = 10<br>Diagnostic centre = 11<br>Others including TG hospital = 12
Hospitalization	Type of hospital	Government hospital / health centre = 1<br>NGO/charitable health centre or hospital = 2<br>Private hospital = 3<br>Others including TG hospital = 4
DOT	Medicine intake under supervision or not	Self-administered = 1<br>Directly observed treatment (DOT) = 2
	Who is the DOT provider?	Health facility = 1<br>Community health worker/volunteer = 2<br>Workplace = 3<br>Family member = 4<br>Others = 5
Picking up drug	Who goes to pick-up drugs?	Patient alone = 1<br>Patient with accompanying Person = 2<br>Household member = 3<br>Friend /neighbour = 4<br>Other (specify) = 5
	How often do they go for drug pick-up?	Every day = 1<br>Every week = 2<br>Every 2 weeks = 3<br>Every month = 4<br>Other = 5
	Where do they go for drug pick-up?	Pharmacy/ Drugstore = 1<br>Dispensary = 2<br>Private clinic/ Private practitioner = 3<br>Quack = 4<br>Government health facility (CHC, PHC, SC) = 5<br>Government hospital = 6<br>Traditional healers/ Herbalists = 7<br>AYUSH (Ayurveda, Yoga, Unani, Siddha, Homeopath) = 8<br>Private hospital = 9<br>Community health worker = 10<br>Diagnostic centre = 11<br>Other including TG hospital = 12

Coping	Did you borrow or receive any money to cover your TB expenses?	Yes = 1; No = 2; Don't know = 3
	From whom did you borrow / receive money?	Family members / relatives = 1<br>Neighbours / friends / colleagues = 2<br>Commercial bank loan (public / private) = 3<br>Cooperative = 4<br>Employer = 5<br>"Unofficial lender" (Black market) = 6<br>Other (specify) = 7
	Are you expected to pay back the amount?	Yes = 1; No = 2; Don't know = 3
	Have you sold / mortgaged any personal belongings for TB treatment?	Yes = 1; No = 2; Don't know = 3
	What did you sell / mortgage?	Land = 1<br>Livestock = 2<br>Transport / vehicle = 3<br>Household items = 4<br>Farm product = 5<br>Gold / jewellery = 6<br>Other (specify) = 7
	Did you or your family withdraw from savings or fixed deposits for your illness?	Yes = 1; No = 2; Don't know = 3
	Did your household do any of these activities because of your illness (TB)	1.Cut down consumption level (Yes / No)<br>2.Other household member started working (Yes / No)<br>3.Withdraw children from school / private tuition (Yes / No)<br>4.Run up an account with a shop (Yes / No)<br>5.Move to other accommodation (e.g. from higher to lower rent) (Yes / No)<br>6.Other (please specify)<br>7.Did not use any of these strategies<br>8. Don't know
	At least once from the time of your TB symptom, were you unable to pay for any of the below listed household items?	1.Electricity bill, gas, mobile bill, cable bill<br>2.Medicines, medical consultations<br>3. Health / life insurance premium<br>4.School / Tuition fees for children<br>5.House rent / shop rent<br>6.Contribution to family / social events (e.g. marriage, festivals)<br>7.Others (specify)<br>8.None (i.e. never missed these payments due to the illness)<br>9. Don't know
