# Supplementary material for: Catastrophic costs for tuberculosis patients in India: Impact of methodological choices
Source: PLOS Glob Public Health. 2024 Apr 26;4(4):e0003078. doi: 10.1371/journal.pgph.0003078 (PMC11051603; doi:10.1371/journal.pgph.0003078)
Supplement: Attachment — Submitted filename: Reviewer comments_2.docx [file pgph.0003078.s008.docx]

**Reviewer comments**

Authors have appropriately addressed most of the comments, and I believe this manuscript is almost ready for publication. Nevertheless, I would like to propose a suggestion:

I acknowledge that authors have used "Proportion of total TB treatment cost in pre-TB annual household income" for assessing the risk of catastrophic expenditure in linear regression. However, may I suggest considering logistic regression model instead of linear regression, with the proportion of households facing catastrophic expenditure as the dependent variable. Logistic regression is also better suited for capturing the impact of independent categorical variables (with multiple categories) such as education, wealth quintiles, etc. This adjustment could provide a better understanding of the predictors influencing the risk of facing catastrophic expenditure.
