# Supplementary material for: Catastrophic costs for tuberculosis patients in India: Impact of methodological choices
Source: PLOS Glob Public Health. 2024 Apr 26;4(4):e0003078. doi: 10.1371/journal.pgph.0003078 (PMC11051603; doi:10.1371/journal.pgph.0003078)
Supplement: Attachment — Submitted filename: Reviewer comments.docx [file pgph.0003078.s006.docx]

**Reviewer comments**

The present study estimates the economic burden (comprising of direct and indirect costs) and associated financial catastrophe among households with a member taking treatment for drug susceptible TB. Two specific methodologies i.e., human capital and output approach, were used to estimate indirect costs. Further six different scenarios were explored to provide a range of households facing catastrophic costs depending upon the type of indirect cost calculation method and the denominator used for the calculation of catastrophic cost. I congratulate the authors in successfully undertaking this analysis. Although, overall, the study methodology and analysis looks great, I have a few comments as follows:

1. The authors have stated in the discussion that it is difficult to comment upon the best method for indirect cost calculation as different methods have different policy implications. I recommend discussing the pros and cons of each indirect cost calculation method. As the present analysis shows a wide range of households facing catastrophic cost, i.e., from 30% to 70%, based on the type of method used for indirect cost calculation, authors need to provide an overall conclusion that which specific approach (may be not the best) is more suitable for indirect cost calculation within the Indian context.

HCA measures the lost productivity but does not capture indirect cost due to reduced productivity (as it just multiplies the time spent while seeking and receiving care with the wage rate). While on the other hand, OA seems to capture the reduced productivity in the form of income loss, but it misses the indirect costs associated with the time spent by those guardians or carers who are involved in unpaid household services (such as housewives). As the authors have stated, I also believe that each approach tries to capture the different components of indirect costs. But, as the main highlight of the study is to assess catastrophic costs using different methods of indirect cost calculation, I suggest authors to include more comprehensive arguments (as above) related to these indirect cost methods.

2. Authors need to clearly state in the methods that whether both direct and indirect cost were included in the calculation of catastrophic costs, as it seems from table 1 that only indirect costs were included.
3. To have a clear picture of the treatment costs, please include a separate descriptive table, either in the main text or in the appendix, showing the component wise breakdown of the direct and indirect cost at different stages of the TB treatment, among the three different sub-groups studied in the present analysis.
4. Please add a title for y axis in fig. 4 and 5. Y axis seems to show the proportion of households facing financial catastrophe. In fig. 4, I would recommend providing a ‘p value’ and showing whether the difference in the extent of catastrophic costs across the income groups was statistically significant or not. Likewise, a ‘p-value’ for showing difference in catastrophic costs across type of TB in Fig. 5 could be mentioned.
5. I could not interpret and fully understand the relevance of Fig. 3. While some of the blue dots suggest that pre-TB annual household income is zero, and the households faced some amount of expenditure in the pre-treatment phase. I am unsure, I am correctly interpreting the figure, but how could annual household income be zero? Likewise, in Fig. 2, some of the dots correspond to a pre-TB annual household income of zero. Please provide more information and explanation in interpreting these figures.
6. The authors mentioned in Table 2 that around 20-30% of the households had some form of health insurance. Authors may provide more details on the type of insurance and which specific services were covered under it. Further, it would be great to see whether the presence of insurance, in comparison to households without any health insurance, led to any reduction in OOPE or catastrophic costs.
7. In addition to univariable analysis, I would suggest including a multiple regression to examine the association or risk of incurring catastrophic costs, with demographic characteristics, clinical characteristics, adverse events, treatment seeking behaviour, income status, residential status, presence of insurance, etc.
