# Supplementary material for: Catastrophic costs for tuberculosis patients in India: Impact of methodological choices
Source: PLOS Glob Public Health. 2024 Apr 26;4(4):e0003078. doi: 10.1371/journal.pgph.0003078 (PMC11051603; doi:10.1371/journal.pgph.0003078)
Supplement: Attachment — Submitted filename: Reviewer comments and Author responses.docx [file pgph.0003078.s007.docx]

Title: Catastrophic costs for tuberculosis patients in India: impact of methodological choices
Manuscript number PGPH-D-23-02005

| Reviewer #1 | Author responses |
| --- | --- |
| General comments: This research estimated the catastrophic expenditure due to TB. The authors tried to estimate through different indirect cost calculation methods. This manuscript addresses a very important issue on catastrophic cost due to TB and it was done in four states Assam, Maharashtra, Tamil Nadu, and West Bengal. Still it is not clear that which method is most appropriate to be used to calculate indirect cost from this study. Overall this is a good study, but the current version is not suitable for publication and I would recommend this version to have a major revision for being accepted for publication. | Thank you very much for reviewing this manuscript and providing very helpful comments. We have addressed all comments and made necessary changes throughout the manuscript. |
| Specific comments |  |
| I would recommend the authors to modify the abstract incorporating the comments given. The authors can modify the sentences which starts with numbers throughout the manuscript. | We have modified the abstract as well as made sure that no sentence is starting with numbers throughout the manuscript. |
| The sentence in the abstract “Studies included in the review were small scale cross sectional, the results of which are difficult to use for policy decisions” has to rephrased. It can’t be emphasized that all small scale cross sectionals studies are difficult to use for policy decisions. | We have modified the sentence. |
| The novelty of this study has not come out well from this current version of the manuscript. There is no mentioning of the salient findings and conclusion of the study. | We have modified the abstract and the manuscript and clearly mentioned the salient features of the study |
| The last sentence in the introduction also needs to be rephrased. | We have modified the sentence |
| How the four states are considered to be representative of India. | For the costing study, national representativeness can be achieved by considering a limited number of states selected using an appropriately stratified sampling design. The WHO Study on global AGEing and adult health (SAGE) sampling design was followed in this context. As a first step, 29 states of India were stratified into six levels of development and six geographic locations. A composite development index was constructed using the following indicators at the state level: Infant mortality rate; female literacy rate; full immunization coverage rate; and per capita income. Principal component analysis technique was used to construct the composite index. Quantiles were then used to categorize the states into six levels of development. Based on the availability of study budget, Assam, Maharashtra, Tamil Nadu, and West Bengal were selected for this study as these states not only represent different levels of development but also cover different regions of the country. Apart from that, Assam, Tamil Nadu, and West Bengal have substantial land area for tea gardens, and Maharashtra, Tamil Nadu and West Bengal are among the top five states in terms of urban slum population in the country.  As the methods of state selection was discussed in detail in another paper, we did not provide detail information in this paper, however, we have given the reference for further information. Please see page 7, line numbers 143-145 in the track changes version of the manuscript. |
| How the people from tea garden areas are more prone to TB; is it defined in the national strategic plan; please check. | Yes, this group is defined as high-risk group in national strategic plan, 2017-2025. Please see Page 23 of the national strategic plan. |
| I recommend the analysis to be done separately for respective population; since urban slum and rural may have more catastrophic expenditure. It is not sampled separately sub group analysis can be done for belter understanding. | We did not conduct analyses by rural and urban, however, all our study participants from tea garden areas were from rural areas while participants from urban slum areas were from urban and participants from general population were from both rural and urban (please see Table 2). Hence, we presented catastrophic costs by different groups. |
| The reason for using different methodologies and the appropriate use of methodologies have not been given. | We have now provided the reasons of using different methodologies and explained the difference among those |
| In terms of indirect cost, the reason for the loss of income is due to TB or something else? How this is collected? Sometimes, the loss of income may be due to unemployment. Further, when patients can go for work, but doctors’ advise them to take rest during the treatment. In this scenario, some might go for work while some may not. Is this accounted in the study? | Income loss reported in this study was specifically for TB. We interviewed each patient 3 times. During 1^st^ interview, we asked them about patient and household income before they had TB and at the time of interview (intensive phase of treatment). In the second interview, we asked patient and household income at the time of interview (these interviews were at the end of treatment). From these data, we calculated loss of income as compared to pre-TB income.  Income loss was primarily because of unemployment, it could be either the patient was unable to work or as the reviewer rightly mentioned, doctors asked them not to work.  All these scenarios were considered in this study. |
| Self-reported income and expenditure was collected for this study. Is any quality control mechanism followed? (For example, any cross checking) | We cross-checked income data collected in different rounds for consistency. However, we did not notice discrepancy in reporting. As mentioned in page 27, lines 494-496 in the track changes version of the manuscript, we find it difficult to collect consumption expenditure data and we did not cross check the data either. |
| The cost for death, loss to follow up and relapse will be high. The current results might be under estimated. This needs to be included in the limitation. | In this study, we calculated cost of treatment for one episode of TB from patient perspective. While we completely agree with the reviewer that the relapse case will have impact on patient, however, that will be after treatment completion. In this study we restricted our analysis till treatment completion. Please see page 7, line numbers 151-153 in the track changes version. Further, effort to bring loss to follow up patients in treatment will be a cost to the government. As we considered patient perspective, we did not consider those costs. |
| For extra pulmonary TB, the diagnostic costs will be very high due to the lack of diagnostic tools. That needs to be discussed elaborately. | We have now discussed this elaborately. Please see page 28, lines 514-524 in the track changes version |
| Authors used two different terms, ‘Discontinuation of treatment ‘and ‘Loss to follow up’. Is this different or same? | To avoid this confusion, we changed ‘Loss to follow up’ to ‘Missed follow up interviews’. By ‘Loss to follow up’ we meant the patients we lost for following up for our interviews, however, we understood the reviewer’s concern and hence, we rephrased ‘Loss to follow up’ throughout the text. |
| The current treatment in India is daily regimen and drugs are supplied to patients. Whether DOT is followed now? | DOT was followed in few TB units and wherever it was followed, we considered travel expenses and time spent for DOT as well. Please see Table 2 Treatment support which shows the proportion of study participants under DOT. |
| The current study changed the denominator for catastrophic cost calculation. How this can be compared with previous studies? | Pre-TB annual household income has generally been used as denominator of catastrophic cost. In our study, we also used the same. Apart from that, as studies have shown that household expenditure is a better proxy, we used pre-TB household annual expenditure as denominator. Hence, we did not change it, we used two different denominators. Therefore, our results using pre-TB annual household income as denominator is comparable with other studies. |
| What is the role of the current intervention in reducing the catastrophic expenditure like NIKSHAY MITRA, ₹500 per month to patients, active case finding rapid diagnostic tools and molecular diagnostic which diagnoses early before patients becomes very sick. Is there any role health insurance in reducing the catastrophic cost since majority have health insurance. | In this paper, we did not examine the role of various interventions in catastrophic cost as this was beyond the scope of this study. However, health insurance did not play any significant role in reducing catastrophic cost as only 21%-34% study participants reported having health insurance (please see Table 2 – Insurance status). We have added this in the conclusion. |
| The TB treatment is completely free. Why patients are borrowing during CP. The reasons can be given. Since it is expected, the patients can become better after IP that they can go for work. Is this borrowing for treatment or something else? | Yes, as the reviewer rightly pointed out that it is expected that patients will get better in CP and will join work, but our study showed many patients were still unemployed during CP and to manage households, they had to borrow money. We have now explained this in coping strategies section. |
| Is it possible to give the factors that contributes to catastrophic expenditure? | We have now provided regression results to identify factors to catastrophic cost. Please see Table 4. |
| In line 252: no trace to be rephrased. | We have rephrased this. |
| In line 340 to 341 needs clarity. | We have rephrased this. |
| In line 351,352, the sentence to be changed to start with words. | We have changed these |
| In line 391-393, 398-400 and 436-437 needs to be rephrased with clarity. | We have modified all sentences |
| It was not estimated the current study costs for MDR, Relapse and lost to follow-up patients; but it was mentioned in the conclusion. Conclusion form the current study evidence. Similarly for insurance and Nikshay Poshan Yojana. | We have deleted the discussions around MDR discontinuation and NPY and only focused on evidence from this study. |
| Reference to be followed the journal style. Reference number 2, 5, 12, 13 and 21 seems to be incomplete reference | We have cross-checked the references and those are fine. |
| Figure axis name is to be uniform (currently, some figure title is in capital letters and some are in small letter) | We have now changed these and made all Figure axes uniform |
| **Reviewer #2** |  |
| The present study estimates the economic burden (comprising of direct and indirect costs) and associated financial catastrophe among households with a member taking treatment for drug susceptible TB. Two specific methodologies i.e., human capital and output approach, were used to estimate indirect costs. Further six different scenarios were explored to provide a range of households facing catastrophic costs depending upon the type of indirect cost calculation method and the denominator used for the calculation of catastrophic cost. I congratulate the authors in successfully undertaking this analysis. Although, overall, the study methodology and analysis looks great, I have a few comments as follows: | Thank you so much for reviewing this manuscript and providing very helpful comments. We have addressed all comments and made all necessary changes throughout the manuscript. |
| The authors have stated in the discussion that it is difficult to comment upon the best method for indirect cost calculation as different methods have different policy implications. I recommend discussing the pros and cons of each indirect cost calculation method. As the present analysis shows a wide range of households facing catastrophic cost, i.e., from 30% to 70%, based on the type of method used for indirect cost calculation, authors need to provide an overall conclusion that which specific approach (may be not the best) is more suitable for indirect cost calculation within the Indian context.  HCA measures the lost productivity but does not capture indirect cost due to reduced productivity (as it just multiplies the time spent while seeking and receiving care with the wage rate). While on the other hand, OA seems to capture the reduced productivity in the form of income loss, but it misses the indirect costs associated with the time spent by those guardians or carers who are involved in unpaid household services (such as housewives). As the authors have stated, I also believe that each approach tries to capture the different components of indirect costs. But, as the main highlight of the study is to assess catastrophic costs using different methods of indirect cost calculation, I suggest authors to include more comprehensive arguments (as above) related to these indirect cost methods. | We have added the suggested changes in the manuscript. Please see pages 8&9, line numbers 173-185 in the track changes version. |
| Authors need to clearly state in the methods that whether both direct and indirect cost were included in the calculation of catastrophic costs, as it seems from table 1 that only indirect costs were included. | We have now added a sentence with the definition of catastrophic cost where we mentioned both direct and indirect cost. |
| To have a clear picture of the treatment costs, please include a separate descriptive table, either in the main text or in the appendix, showing the component wise breakdown of the direct and indirect cost at different stages of the TB treatment, among the three different sub-groups studied in the present analysis. | We have now added a new Table with detailed cost break-up. Please see Table 3. |
| Please add a title for y axis in fig. 4 and 5. Y axis seems to show the proportion of households facing financial catastrophe. In fig. 4, I would recommend providing a ‘p value’ and showing whether the difference in the extent of catastrophic costs across the income groups was statistically significant or not. Likewise, a ‘p-value’ for showing difference in catastrophic costs across type of TB in Fig. 5 could be mentioned. | Thank you so much for pointing these. We have now added axis titles in Figures 4 and 5 and added ‘p-value’ in both Figures . |
| I could not interpret and fully understand the relevance of Fig. 3. While some of the blue dots suggest that pre-TB annual household income is zero, and the households faced some amount of expenditure in the pre-treatment phase. I am unsure, I am correctly interpreting the figure, but how could annual household income be zero? Likewise, in Fig. 2, some of the dots correspond to a pre-TB annual household income of zero. Please provide more information and explanation in interpreting these figures. | Fig 3 tells us among those who faced catastrophic cost, proportion of patients faced the cost before starting the treatment and proportion faced during treatment. Also, the trend of costs incurred during treatment phase was linear while the same was scattered during pre-treatment phase.  To respond to your query on zero household income but still faced catastrophic cost, we had 18 study participants who had zero household income. This could be because they were old and living using their savings, or they were living alone with no income and were taken care of friends/family members/neighbours who were not part of the household. During their sickness, they were taken care of by their relatives who spent money and time, however, as they were not part of the household, household income was zero. Hence, for some of these study participants, as household income was zero, any cost incurred during TB treatment was catastrophic.  We have now mentioned this in the text for clarity. Please see page 18, lines 351-357 for the explanation. |
| The authors mentioned in Table 2 that around 20-30% of the households had some form of health insurance. Authors may provide more details on the type of insurance and which specific services were covered under it. Further, it would be great to see whether the presence of insurance, in comparison to households without any health insurance, led to any reduction in OOPE or catastrophic costs. | We have now added types of insurance and how it was used. Only 3 out of 1482 study participants received reimbursements for hospitalization through health insurance, hence, it had no impact in reducing catastrophic cost. We have mentioned that in the conclusion. |
| In addition to univariable analysis, I would suggest including a multiple regression to examine the association or risk of incurring catastrophic costs, with demographic characteristics, clinical characteristics, adverse events, treatment seeking behaviour, income status, residential status, presence of insurance, etc. | We have now added regression analysis. Please see Table 4. |
